# Supplementary material for: Evaluating lubricant performance to reduce COVID-19 PPE-related skin injury
Source: PLoS One. 2020 Sep 24;15(9):e0239363. doi: 10.1371/journal.pone.0239363 (PMC7514078; doi:10.1371/journal.pone.0239363)
Supplement: S1 File — (PDF) [file pone.0239363.s001.pdf]

Science Engineering Technology Research Ethics Committee
Imperial College London
Room 221
Medical School Building
St Marys Campus
London
W2 1PG
Tel: +44 (0)207 594 1872

researchethicscommittee@imperial.ac.uk

19 May 2020

Dear Dr Marc Masen

Study Title:. Skin friction measurements to assess efficacy of lubricants

SETREC reference: 20IC5999

The above study was approved by your Head of Department on 12/05/20 and by the Joint Research Compliance Office on 19/05/20.

Under the Science Engineering Technology Research Ethics Committee process, a study that has been reviewed by the Joint Research Compliance Office and Head of Division/Department, where no significant ethical issues have been identified in the protocol or ethics application, can be given JRCO approval without requiring it to go to full committee.

Documents
The documents reviewed were:

- Application form (v3.0 19/05/2020)
- Protocol (v3.0 19/05/2020)
- Consent Form (v2.0 18/05/2020)

Yours sincerely,

Ruth Nicholson

Digitally signed by Ruth Nicholson
Date: 2020.05.19 13:44:22 +01'00'

Ruth Nicholson,
Head of Research Governance and Integrity,
Imperial College London
