# Supplementary material for: Evaluating lubricant performance to reduce COVID-19 PPE-related skin injury
Source: PLoS One. 2020 Sep 24;15(9):e0239363. doi: 10.1371/journal.pone.0239363 (PMC7514078; doi:10.1371/journal.pone.0239363)
Supplement: Attachment — Submitted filename: Response to Reviewers.docx [file pone.0239363.s002.docx]

We would like to thank the reviewers for their positive comments and the helpful suggestions. Please find below our responses to the points raised, which we have also implemented into the manuscript. Changes to the manuscript have been marked in red font.

Reviewer #1: Healthcare workers around the world are experiencing skin injury due to the extended use of personal protective equipment (PPE) during the COVID-19 pandemic. This study aims to provide a practical lubricating solution for frontline medical staff working a 4+ hours shift wearing PPE.
 A literature review into skin friction and skin lubrication was conducted to identify products and substances that can reduce friction. We evaluated the lubricating performance of commercially available products in vivo using a custom-built tribometer. The results can improve our understanding of evaluating lubricant performance to reduce COVID-19 PPE-related skin injury. The detailed comments are presented below:

1. The study was approved by the Imperial College London Science, Engineering and Technology Research Ethics Committee (SETREC), reference 20IC5999. Please provide evidence.

*The decision letter from SETREC evidencing approval has been uploaded with the revised document.*

2. In this study, the experimental programme was performed over a one month period, from mid-May to mid-June 2020. Please list your work for each period in detail

*Tests were limited to maximum two lubricating substances per day throughout this entire period, to prevent affecting the results. A three-hour resting period was used between experiments. In the manuscript this has been added:*

*If a test provided a much-elevated friction compared to other products in the same category, then further tests using this product were not conducted. Additionally, if a test showed a strong increase of friction with time, no further measurements at other time-intervals were performed. After each experiment, the product was removed from the skin using a gentle wipe with a paper tissue and cleaned using water and non-soap body wash, followed by a recovery period of at least 3 h before another experiment was commenced.*

3. In this study, there are some formulas. But the formula is not numbered. Besides, each variable needs to be explained.

*The equations 1 and 2 have now been numbered. The text above eq. 1 has been adapted to clarify the meaning of the variables. The text now reads: The total friction response is the combination of the forces related to viscoelastic deformation of the bulk material,* $F_{bulk}$*, and the forces related to breaking of intermolecular bonds on the interface between the two materials,* $F_{int}$ *(eq.1). Adams et al. demonstrated that for contacts between skin and other surfaces the interfacial friction dominates the total frictional effects, whilst viscoelastic effects may be ignored:^23^*

| $F_{f}=F_{bulk}+F_{int}\approx F_{int}$ | ***eq. (1)*** |
| --- | --- |

4. There are so many authors for this article. A statement for your authors' contribution and conflicts of interest are necessary.

*The statement of author contributions, as well as a declaration of conflicts can be found at the bottom of the document.*

Reviewer #2: This is a very interesting experiment. In this study, a 44 year old caucasian man was recruited to test the effectiveness of a series of lubricants to reduce friction and shear force in the left forearm (instead of facial skin), and hope find which lubricants has a protective effect for more than 4 hours. However, the experimental design did not consider the following factors:

1.Participants: The subjects' health status was unknown, such as height, weight, etc. Only one caucasian male was selected and does reflect the friction and lubricant effect of women and other skin color health care workers on their skin ?

*The subject’s health status has been added to the document: “Due to COVID-19 restrictions, performing in vivo tests was limited to a single participant. The participant, Caucasian male, healthy, age 44 years, mass 80 kg, height 1.84 m, gave informed consent.” In addition, effects of skin variations (as far as they are known from literature) are discussed in the Participants section. To further clarify, in the limitations section we have added the following sentence: “Whilst quantitative results vary between people, literature shows that qualitative trends in friction are repeatable.^16, 20, 21^”*

2.Setting: The volar forearm was selected as the experimental site. Does affect the friction coefficient measured after using the lubricant due to the different thickness of between the stratum corneum on the volar forearm and the facial skin? Does affect the friction coefficient measured after lubricant use due to different perspiration of facial skin and forearm skin? These are both addressed in the limitations section but can be expanded upon further as appropriate? In this study, authors measured the static friction coefficient , while the skin of medical staff wearing PPE against covid-19 produced dynamic friction coefficient. How to explain the correlation between the two? In the fight against covid-19, the skin damage caused by wearing PPE was related to the increased friction between skin and PPE caused by pressure, rapid exercise and sweating. Does consider the increase of friction force under multi-factors when lubricant is used to reduce friction?

*The reviewer makes several good points. Hendriks and Franklin (22) discussed the differences between facial skin and forearm skin – their conclusion was actually more firm that we initially reported in our manuscript. They state: “There is no significant difference between the overall average friction coefficient measured on the cheek and the ventral forearm.” We have updated this in the manuscript.*

*As discussed in the manuscript, perspiration is an interesting variable that needs further investigation. To keep our investigation manageable, in this study the effects of perspiration were limited as much as possible, because perspiration characteristics between people vary strongly: Perspiration may not only affect the lubricant, but also the mechanical behaviour of skin, the skin surface as well as the PPE surface. Whilst the initial response would most likely be a strong friction reduction, the potential longer-term effects of perspiration include a strong increase in friction, meaning people who perspire strongly may need extra focus on a skin care regime. This requires a more in-depth study with a large cohort, which is well outside of the scope of our investigation.*

*Regarding the static and dynamic friction: the main driver for skin bruising is the combination of pressure and shear, see e.g. refs 7, 11 and 14. The shear can be caused by static friction (‘pulling’/’gripping’) but also by dynamic friction (‘slipping’/’sliding’). Whilst dynamic friction will have an effect, the type of injury usually related to dynamic friction are blisters and abrasions. Nevertheless, the reviewer is correct that the (macroscopic) motion of the PPE may have an effect. Veijgen [16] studied the relationship between the static and dynamic friction response of a large cohort and found that the two are directly correlated.*

3.Experimental products: The author selected four kinds of 19 different products (Table 1 on page 7-8) to be used in the same part of the same person in a month. The friction coefficient of each product was measured three times at 1, 2 and 4 hours after use. Will the frequent use of different products change the microclimate of the skin and thus affect the friction coefficient?

*The reviewer makes a fair point, which indeed needs clarification in the manuscript. Not all products were tested for 1, 2 and 4 hours. We updated to protocol section: “If a test provided a much-elevated friction compared to other products in the same category, then further tests using this product were not conducted. Additionally, if a test showed a strong increase of friction with time, no further measurements at other time-intervals were performed. “* *After each experiment, the product was removed from the skin using a gentle wipe with a paper tissue and the skin was cleaned using water and non-soap body wash, followed by a recovery period of at least 3 h before another experiment was commenced. A friction measurements on untreated, or ‘dry’ skin was performed at the start of every test day and, although dry friction results are inherently somewhat more variable than lubricated experiments as evidenced by the error bars in Fig. 3, no deviating trends were observed. This indicates that there was no significant change in the friction behaviour of the skin during the course of the experimental programme.*”

Analysis of the results and discussion of this study has the following doubts:

1. Figures 4A to 4F showed schematic rather than measurement results. What does the author want to tell the readers? Figures depict the theorised driving mechanisms behind the friction on the skin and the lubricants used. These aren’t measurement results and are rather and explanation of the prior results shown in Figure 3.

*The reviewer is correct, all obtained results are presented in Figure 3. The schematics of figure 4 serve to discuss the underlying mechanisms. This is highly relevant as it will provide readers with further insight into the various mechanisms, which will help in the development of improved products that are tailored for shear reduction, and not just moisturising or hydrating, which most commercially available creams appear to be.*

2.In the discussion, the authors inferred the results based on these diagrams. Can they reflect the real situation?

*The real situation is depicted in Figures 3, which shows our results. The purpose of figure 4 is to facilitate the discussion. For this we need simplified diagrams hypothesizing on the various mechanisms. We see this as ‘as close to the real situation’ as we can get.*

3.The authors has come to the following conclusion: Results indicate that the use of emollients and moisturizing creams is to be discouraged when wearing PPE for long duration as they may result in excessive shear forces acting on the skin. Talcum powder, a lanolin containing petrolatum, and a coconut oil-cocoa butter-beeswax mixture provide excellent long-lasting lubrication. But there seems to be little evidence to support.

*Our results provide the evidence in terms of experimental results that the three listed products provide enduring low friction, thus reducing the shear loading on the skin. In contrast, excessively high friction was obtained for several moisturing-type cosmetics.*

Minors
 1.There are some spelling mistakes in the text.

*We corrected the spelling mistakes we found.*

2.The author described it in the abstract: This research was funded by the Imperial College COVID-19 Response Fund. But on page 10 it was written: The research funder did not play a role in the research. What does that mean?

*This sentence was added to emphasize that the funder did not have any influence over the decisions made in the research. We have clarified this by changing the sentence to: “The research funder did not play a role in the execution of the research.”*

Reviewer #3: Very interesting and relevant study looking at lubricants to protect skin health in those using PPE during COVID-19. I have some minor points which require clarification.

1. Friction is only one element of the boundary conditions that causes skin damage. Pressure, shear and an altered microclimate will also effect skin health. This is introduced in the manuscript but perhaps a little miss-leading in figure 1. If the same pressure and shear is applied to the skin, its likely to cause similar tissue deformation regardless of lubricant.

*The reviewer raises an interesting point about skin mechanics and the complexities that occur after lubricants are introduced. The aim of Figure 1 (B) is to illustrate the extent of damage caused by PPE leading to the clinical problem that is faced by frequent PPE wearers. We have significantly reduced the size of the deformation in Figure 1B and have amended reduced the size of the blue shear force arrow in Figure 1 (C) to clarify the situation.*

*Our article suggests a variety of mechanisms in which lubricants affect the PPE-skin system, as illustrated in Fig. 4 of the main article. Therefore, we cannot disregard the effect of the lubricant in this dynamic biological scenario. We are suggesting that the PPE-skin system mechanics will change as a result of lubricant addition.*

2. In addition, Figure 1 should include ‘pressure and shear induced skin injury’ – shear will not be acting in isolation, unless you are referring to tissue shear strain.

*The reviewer is correct that all these effects matter. The main issue is that the combination of pressure and shear has a much larger effect on tissue deformation than pressure alone. Shear stresses are highly efficient in deforming soft materials and therefore causing injury in tissue. Figure 1 shows both the pressure (red arrow) and shear (blue arrow). The text in the figure has been updated to pressure and shear induced skin injury.*

3. Did you recreate the material interface of PPE devices when assessing the friction properties? You discuss the implications of skin epidermal absorption, occlusion and water retention, but does this also apply for the PPE interface material?

*The skin-material interface mimics the interaction properties in terms of contact pressures, and materials used. Like most PPE surfaces, the used PDMS specimens did not absorb moisture.*

4. In the introduction it would be worth highlighting PPE devices which are typically implicated in damage, for example respiratory protective equipment (FFP3 masks)

We agree and have updated the first sentence of the manuscript, which now reads: “*Personal Protective Equipment (PPE) such as goggles, visors and respiratory protective equipment are a critical component in the defence against infectious diseases for frontline medical personnel.*”

5. NHS England and NHS Improvement [4] do not recommend petrolatum to the skin. This has been reported in one manuscript with limited evidence for its efficacy.

*The reviewer makes a fair point here, which is partially due to the referencing style at the end of the sentence. Whilst the NHS does not recommend using petrolatum, they advise to “keep your skin clean and well hydrated/moisturised – apply creams at least 30 minutes before applying PPE. Consider use of a barrier skin wipe/skin protectant if you are likely to be wearing PPE for extended periods.” The sentence in the manuscript has been modified to: This is also recognised in recent literature and advice to medical staff, which include half-hourly application of a lubricant or moisturizer to the skin.^4,14^*

6. Many PPE devices that are implicated in skin damage e.g. the aforementioned FFP3 masks do not employ PDMS at the interface.
 <https://www.3m.co.uk/3M/en_GB/company-uk/3m-products/~/3M-Aura-Disposable-Healthcare-Respirator-FFP3-Valved-1873V-/?N=5002385+3292799385&rt=rud>

*The key characteristic with the materials used to interact with the skin is that they compliant (soft), in an effort to reduce the pressures. However, the associated increase in contact area by such soft materials causes increased friction. Whilst it indeed would be interesting to also assess the material aspects to improve the contact characteristics, this would mainly apply to non-lubricated contact, when the material is in direct contact with the skin. In this study the objective was to provide effective lubrication, meaning the exact material of the PPE is not critically important.*

7. Brill et al. [17] evaluated the pressures required to secure non-invasive ventilation masks, this may differ to the pressures observed in PPE equipment.

*We agree that ventilation masks are not the same as PPE, and that Brill’s work aimed at sealing. However, many PPE such as FFP3 masks are also sealed tightly with the skin, as the reviewer also states in their final point, below . Brill’s values provide a reasonable estimate that can be readily applied.*

8. I appreciate the forearm is convenient for testing, however does this replicate the skin properties of the face, for example the highly porous sites at the nose? As you’ve highlighted there are large differences in friction for unlubricated skin, which are related to variations in sebum, hydration and mechanical properties

*See our answer to point 2 raised by reviewer 2*

9. Strip waxing the forearm would remove layers of the stratum corneum and result in increased transepidermal water loss. Could this have effected your results? Why not use a commercial hair removal cream?

*There are indeed various ways to remove the hair from the skin. Three options were considered: shaving, waxing and chemical removal. Shaving leaves stubble and was therefore discounted, whilst the potential side effects from chemical removal, which include potentially long-lasting effects such as allergic reactions and burns, were deemed excessive and potentially disruptive to the research plan. Andrews et al (AAPS PharmSciTech. 2011 Dec; 12(4): 1393–1400) indicate that the structure and barrier function of the epidermis recover within 12-24 h after stripping, so stripping/waxing with a 2-day waiting period this was considered the best option.*

10. Did you measure the biophysical properties of the skin/check for blanching erythema? Seems like the single participant was put through a large number of mechanical tests.

*We did not have access to any additional equipment during lockdown. In addition, whilst the participant was indeed put through a lot of tests, in terms of actual mechanical loading of the skin, a single experiment took about 10 s, meaning the skin was gently loaded for up to 5 minutes per day. So the total loading of the skin was quite mild and benign.*

11. In-between tests the PDMS was applied, so the interactions between skin and PDMS under mechanical loading conditions was not tested? - you have addressed this to some extent in the discussion

*Indeed, a PDMS specimen was placed onto the skin and secured using plaster. Whilst the loading on the skin is not exact, it enabled some simulation of the contact and the breakdown characteristics of the skin-ppe contact*

12. Results are very descriptive, some inclusion of data, or for example percentage change values from baseline would add context for the reader.

*Percentage changes in terms of value compared to the baseline have been added in red font to the results section. As the results section is quite long, we have not included it here.*

13. No mention of the discussion regarding the functionality of PPE. Indeed, FFP3 masks must retain a seal with the skin in order to filter >95% of particles. Any additional sliding at the interface could be to the detriment of the devices functionality. There must be a careful interplay between maintaining skin health and PPE function.

*We agree that the functionality of PPE is a balance act. Based on our results, we can provide advise towards the effects of lubricants on the reduction of shear on the skin, but it is difficult to comment on the functionality of PPE. In the discussion we have added the sentence: “Finally, care should be taken that the use of lubricant does not affect the functionality and sealing capacity of the PPE.”*
